# Difficult Cases of Extraction

**Published:** 1855-01

**Authors:** James Taylor


### DIFFICULT CASES OF EXTRACTION.

BY DR. JAMES TAYLOR.

The series of articles by Dr. C. T. Cushman, headed "*Practical Thoughts on Tooth Drawing*," the last of which we give in this number of the Register, bring to mind several cases of a similar character, and as we regard the detail of practice as of the first importance to the Profession, we have concluded to give a few of these cases as rather in connection with Dr. Cushman. With Dr. Cushman, we regard the Key, at times, and in certain cases, as an invaluable instrument, and we fear that, at times, much unnecessary pain is inflicted by some in the Profession merely because they regard it unprofessional to use this instrument. We now give a case in point. Some few weeks since, an old patient called on me, with violent pain in the first molar of the left side Inferior Maxillæ. From the nature of the pain and the appearance of the tooth, I soon perceived it to be a severe case of Periodontitis. On examination, I found the tooth much decayed, and on its palital face, broken down to the aveolus, positively affording no resting place for the inner beak of the forceps. The labial portion of the tooth appeared tolerably strong, and with my gum lancet I thoroughly tested its density by cutting and scraping its decayed portion. When I had thus satisfied myself of its true condition, I remarked, "I thought I could remove it with the key without any difficulty." To the use of this instrument she stoutly objected, and as I had fully set my thoughts on the key or nothing else, I demurred to the use of the forceps. Thus we separated. The subsequent history, as I had it from a friend, was, that she went to an experienced operator, of this city, and with the first application of the forceps he broke the tooth, and after many repeated efforts, consuming half an hour, the roots were removed, but not until the lady had fainted. She was taken home in a carriage, and did not leave her bed for several days.

This tooth, it may be said, might have broken with the key, and so it might, yet in my hands, I know that the key would have been the safest instrument. This was a case where I regard the key invaluable. I can not, however, agree with Dr. Cushman in the application of the key to the root of the cuspid tooth, as reported in case No. XV, and also that of case No. XVI. For such I use the forceps—beaks, lancet pointed. That instrument which most expeditiously, easily, and certainly removes a tooth is the best. Yet it must be remembered that use and tact will make an instrument in the hands of one operator far more effectual than in the hands of another.

I believe with Dr. Bridges, that "we sometimes encounter teeth that we can not by fair means remove," and yet we may by foul. The history of the following case is illustrative, and we leave our readers to decide by what means success was obtained. Mrs. S——, about two months since, came into my office, with a very troublesome left dens sapientiæ. The tooth wanted room for eruption, and although nearly through, pointing slightly outward, yet it had carried so much of the soft parts up with it, that every effort at occlusion of the jaws, these parts came in contact with the tooth above, and thus inflammation set in. The face was much swollen, and the mouth could not be opened so as to reach the tooth with a proper forcep. The tooth had been ineffectually tried before she came in. This had not effected a loosening of the tooth, yet increased the pain and swelling. With my elevator (Physic's) I loosened the tooth and slightly raised it in its socket, then with an elevator of a different kind applied between this tooth and the posterior molar, I forced the tooth backward, and upward until two lines at least of space was obtained between these teeth. I then applied a curved hawk's bill forcep, and made upward and outward traction. But, from the impossibility of making a proper application of force, backward, the effort was unsuccessful; the tooth merely slipped back into its old position. The same operation, with elevator and forceps, was renewed again and again, and every other means tried which seemed possible, and although the tooth became so loose that it could be moved backward and inward and outward a little, yet it would *not come.* After half an hour's hard labor we both concluded to quit.

The loosening of the tooth abated the violence of the pain, and I told her *that, I thought*, in a few weeks the tooth could be removed.

November, 1854—Mrs. S. called, and on examination, I found the first part which had so much covered her tooth removed by the suppuration which had taken place around her tooth; the swelling had in a great measure subsided, and she could open her mouth a little wider; yet she has suffered more or less ever since I first attempted to remove her tooth. On examination, I found the tooth firmly set. But, as I supposed the processes must be partially absorbed, I told her,

"*I thought*" I could remove the tooth, and set the next day to attend to it.

November, 1854—Mrs. S. called, and with the elevator, (Physic's,) I loosened her tooth, and raised it almost on a line with the posterior molar. I then applied the hawk's-bill forceps, and made outward and upward traction. The tooth resisted all the force I dare apply. I then, with the curved bar elevator applied between this tooth and the posterior molar, (making this and the aveolus as a falcrum for my lever,) tried to force the tooth backward and upward. This was effectual in only gaining a little more space between the teeth, and raising the tooth very little. I then applied the same forcep again, but the tooth moved not. I then tried my curved root forcep, but the mouth could not be opened wide enough to thus reach the tooth. I then applied my straight root forceps, and by grasping the tooth obliquely, I made inward and outward traction. The tooth in the mean time raised so much that the mouth could not be closed.

I re-applied different forceps again and again, and shook, and "wrenched and turned" the tooth in every possible way, and yet the tooth refused to leave its socket. This was continued until my arm gave out and required rest as well as my patient. The fact is, we both felt disposed to quit, but the mouth could no more be closed, and it became necessary to force the tooth back into its place or take it entirely out. I concluded to make one more effort to remove the tooth, and crossed the street to the dental depot of Dr. J. M. Brown, to see if I could find among his extensive assortment a pair of forceps which would grasp the tooth more favorably than any I had. I selected one which is small in the bar and not so much curved as those I generally use for the lower teeth, and which I thought would allow me to make more powerful traction inwardly than the straight pair I had used. Fortunately, the rigidity of the muscles had somewhat given way, from the long strain upon them in my efforts to remove the tooth, and I was enabled to grasp the tooth with this forcep, and after a great deal of traction inward and outward, and upward, the tooth came out. I never *labored* half so much on any tooth as this- The roots slightly curved backward as I anticipated, but they were not only nearly twice as large in diameter as I supposed, but they were also very much lengthened by exostasis. I know not now how I could have extracted the tooth any better. With any ordinary patient I never should have succeeded. She has just that courage which appears now so necessary in the siege of Sebastopol.

Only a few days before this operation, I had one of a like character, except that the roots were not enlarged by extostosis, and was removed by three or four efforts with the elevator and forceps, but which had withstood the most strenuous efforts of the forceps, unaided by the elevator.

I give another case of a dens sapientiæ which forced me into a different mode of practice. During last winter, a gentleman of Fulton called with severe pain in the neighborhood of the right inferior dens sapientiæ. On examination, the teeth were sound and very firmly set. The integuments around the teeth had commenced to swell, and some rigidity of the muscles existed so as to prevent the mouth from being well opened. The anterior cusps of the dens sapientiæ pressed against the neck of the posterior molar and the large and bulging crown of this tooth appeared to pinion the former to its place; the pain and general symptoms indicated a protracted case of alveolar abscess, resulting from pressure, and owing to its location, one of the worst character. No certain remedy could I prescribe but the extraction of the tooth.

I first applied Physic's Elevator, but it failed to raise the tooth; and, indeed, from the position of the teeth, I feared that I was merely forcing the tooth back into its own socket. I next selected my curved bar elevator and tried to force its sharp point between the posterior molar and the offending tooth, but it was impossible to get below the crown of this organ.

Here was a dilemma. Yet there was one method of relief, and this, with the consent of my patient, was speedily adopted. I took out the posterior molar, and then with a hawk's-bill forcep extracted the dens sapientiæ. This I removed because of the inflammation in its socket, and because it presented its posterior face, instead of its grinding surface, to its antagonizing organ. Here was a case where the pressure could not be relieved by the file, because it was below the gum. I believe it was the first and only case where I have had to take out but the posterior molar tooth to remove the dens sapientiæ.

The following case, I presume, would have been treated with the turnkey, by Dr. Cushman, and, I have no doubt, successfully; but I dislike so much to apply the fulcrum externally, that I determined to try the forceps.

A lady of Covington, some two weeks since, was sent to me, by her physician, who had broken the anterior left inferior molar, and the decay having taken place on its labial face, it broke on this side down to the level of the alveolus, leaving a firm portion above the alveolus on its palital face. She had endured so much pain in the trials to extract the tooth, and the pain become so severe, that she determined to take chloroform and have it out. I administered chloric æther to anesthesia, and with my left inferior molar forceps (which is made with a beak to pass between the roots) I grasped the gum and alveolus over the union of the roots, on their labial portion, forcing the inner beak of the forceps low down; with an inward and upward pull—the edge of the porcess I had enclosed chipped off, and the roots both came out. The amount of gum removed was not half equal to that which would have been bruised and torn up by the fulcrum of the key.

### THE USE OF THE GUM LANCET.

The following case shows the use of the Gum Lancet, and how easily one dentist may give up a patient to another: A few days since Mrs. B—— called with violent pain in the posterior superior bicuspid of the left side. She had been the day before to her dentist to have it removed, but on examination he found it a mere shell, and broken to the gum on its lingual face. The prospect for extraction looked bad. He filed off the outer portion to the gum, and told her it should not be removed until after abscess formed, as he would have to cut away the process, &c. After a wakeful and restless night she called at my office and wished to know if I could take out the root. I told her I thought it could be extracted. I have a pair of Gum Lancets, which are made slightly concave on one side, and convex on the other. They are thus adapted to the roots of the teeth, somewhat as the Forcep blades are. In lancing the gum the point of the blade is forced a little into the socket, and by a leverage force, the root is often loosened, and space is made for the point of my forcep blades—and these should be made very slender and sharp at their points. In this case, after the gums were thus cut, and the root slightly moved, I applied a root forcep, and, by carefully *shaking* the root, it gradually raised in its socket until I felt at liberty to close my forceps enough to permit traction to be made in the root; but this effort, in this instance, broke in the lingual portion of the root, but not, however, until it was so much loosened that I could, by using the outer beak of the forcep as an elevator, remove the root. A little tact in this way—changing the mode of using an instrument—will often insure success, when failure otherwise would be the result.

A case of this kind occurred a year or two since, in the Dental Infirmary. I had given the patient chloroform for the extraction of four or five roots, and a superior posterior molar. With a root forcep I had extracted all the roots, and as the effect of the chloroform was passing off, I hurriedly seized a molar forcep, and at the first effort the lingual portion of the tooth broke down to the alveolus. Without the removal of my forcep I changed the mode of applying force, and pushed the tooth from its socket as with a punch or elevator. Perhaps there are but few of our old operators that are not in the habit of doing the same, yet I name this for the benefit of those just commencing.

I might extend this article in giving difficult cases of extraction, for, like other *troubles*, they have not recently come singly, having had more cases of this kind, in the last three months, than in a year previous. Three or four years since I had a like run of diseases of the antrum; I think it nearly a year since I have met with a case.

Is it ever best to wait the formation of an abscess before extracting a tooth, if it is at all practicable to remove it before? The reverse has ever been my practice; First, because it is the most effectual method of letting out the matter; and second, although the operation is more painful, yet an *immense* amount of pain is avoided, which almost always accompanies the formation of alveolar abscess, and which must necessarily be endured if the tooth is not removed.

